# Hepatic Encephalopathy

**Published:** 2003

**Authors:** Roger F. Butterworth

**Affiliations:** Roger F. Butterworth, Ph.D., D.Sc., is scientific director of the Neuroscience Research Unit at the CHUM (Hôpital Saint-Luc), and professor of medicine at the University of Montreal, Montreal, Canada

**Keywords:** hepatic encephalopathy, toxic drug effect, neurotoxicity, alcoholic liver cirrhosis, symptom, diagnosis, brain damage, impaired balance and coordination, ammonia, manganese, neuroimaging, positron emission tomography, magnetic resonance imaging, drug therapy, dialysis, liver, organ transplantation

## Abstract

Hepatic encephalopathy (HE) is a brain disorder caused by chronic liver failure, particularly in alcoholics with cirrhosis, which results in cognitive, psychiatric, and motor impairments. In these patients, the number of functional liver cells is reduced, and some blood is diverted around the liver before toxins are removed. As a result, toxins such as ammonia and manganese can accumulate in the blood and enter the brain, where they can damage nerve cells and supporting cells called astrocytes. Positron emission tomography analyses have determined that ammonia levels are elevated in the brains of HE patients; ammonia accumulation can alter the expression of various important brain genes. Magnetic resonance images show that manganese is deposited in a brain area called the globus pallidus; manganese deposits may be responsible for structural changes in the astrocytes that are characteristic of HE. Treatment of patients with HE involves measures to lower ammonia levels in the blood, medications to counteract ammonia’s effects on brain cell function, devices to compensate for liver dysfunction, and liver transplantation.

The liver and the brain interact in numerous ways to ensure normal brain functioning. For example, the liver plays a key role in supplying nutrients to the brain, which cannot produce these compounds itself. The liver also removes toxic substances from the blood, including substances that have been generated in the brain and must be eliminated from the body, as well as compounds produced in other tissues that are harmful to the brain’s nerve cells (i.e., are neurotoxic). Thus, liver dysfunction can cause disturbances of brain function and even contribute to brain damage.

Liver dysfunction of varying severity is a frequent complication of chronic alcohol abuse. The most common and least severe form of alcoholic liver disease—fatty liver (steatosis)—is characterized by fat deposits in the primary liver cells (i.e., the hepatocytes). More serious stages of alcoholic liver disease include inflammation of liver tissue (hepatitis), scar tissue formation (fibrosis), and destruction of the normal liver architecture (cirrhosis). When the liver becomes fibrotic and cirrhotic, the number of functional hepatocytes decreases, and the liver loses its capacity to remove toxic substances from the blood. Moreover, during these disease stages some of the blood entering the liver through the portal vein cannot penetrate the diseased liver and is diverted directly into the general circulation; this phenomenon is known as portal-systemic shunting. Blood that bypasses the liver is not detoxified, and blood levels of toxic substances increase. Researchers have identified several toxins that normally are removed in the liver but are found in the circulation of patients with alcoholic cirrhosis, including ammonia, manganese, and chemicals called mercaptans, all of which readily enter the brain and are neurotoxic. Consequently, brain function in patients with severe alcoholic liver disease is compromised, resulting in a condition known as hepatic encephalopathy (HE) or portal-systemic encephalopathy.^1^

This article describes the characteristics and diagnosis of HE and the changes in brain cell structure associated with this condition. The article also reviews imaging techniques that allow researchers to study changes in brain structure and function occurring in patients with HE and describes the contributions of ammonia and manganese to the development of HE, as elucidated by these techniques. Finally, the article explores some approaches currently used or being investigated for treating patients with HE resulting from alcoholic liver disease.

## Characteristics and Diagnosis of Hepatic Encephalopathy

HE is a complex neuropsychiatric syndrome characterized by severe cognitive, psychiatric, and motor disturbances resulting from chronic liver failure, which in most cases in Western societies is caused by chronic alcohol abuse. As chronic liver failure develops and increases in severity, patients start to experience sleep disturbances, changes of mood and personality, and a shortened attention span. These symptoms are followed by psychiatric conditions such as anxiety and depression, as well as by motor problems, including motor incoordination and a type of flapping tremor of the hands called asterixis. Ultimately, patients no longer respond to external stimuli and may fall into a coma (i.e., hepatic coma), which can be fatal.

A fact largely overlooked in the literature on alcoholic brain disorders is that patients suffering from HE often have other alcohol-related brain disorders as well, such as Wernicke’s encephalopathy and alcoholic cerebellar degeneration (Kril and Butterworth 1997). These conditions are caused by alcohol-induced deficiencies in the vitamin thiamine and/or alcohol’s direct toxic effects on the brain. It can be difficult to disentangle the contributions of concurrent alcohol-related brain disorders on the patient’s cognitive functioning; however, when an alcoholic patient develops significant liver disease, HE becomes a major factor contributing to the cognitive dysfunction associated with chronic alcoholism. This is clearly illustrated by the results of studies involving neuropsychological tests, which demonstrated that alcoholic patients with cirrhosis (who therefore could be expected to have HE) had significantly lower scores in learning and memory tests than did alcoholics without cirrhosis (Tarter et al. 1993).

HE is difficult to diagnose in alcoholic patients because no single clinical or laboratory test is sufficient to establish the diagnosis. Patients frequently are misdiagnosed, particularly in the early stages of HE, when symptoms occur that are common to a number of psychiatric disorders, such as euphoria, anxiety, depression, and sleep disorders. The following characteristics have been proposed as helpful in diagnosing HE in alcoholic cirrhotic patients (Tarter et al. 1993):

- History of liver disease.
- A slowing (i.e., reduced frequency) of brain waves measured by electroencephalography (EEG).
- Impaired performance on neuropsychological tests assessing visuospatial and perceptual–motor control (e.g., line-drawing tests and number connection tests).
- Asterixis (“flapping tremor”).
- Foul-smelling breath associated with liver disease (i.e., fetor hepaticus).
- Enhanced rate of breathing (i.e., hyperventilation).
- Elevated concentrations of ammonia in the serum^2^ after a period of fasting.
- Reduced awareness or consciousness.

Although these signs and symptoms are useful tools for diagnosing HE, they also occur in brain disorders caused by metabolic disturbances and therefore are not specific to HE. Furthermore, whether—and to what extent—a patient displays each symptom may vary depending on fluctuations in the patient’s medical and drug status or diet. For example, sedatives or meals high in protein frequently trigger episodes of HE in alcoholic patients with cirrhosis. Thus, an asymptomatic cirrhotic patient frequently begins to show signs of HE following ingestion of large amounts of protein, which is a source of ammonia. Also, small doses of sedatives may trigger overt encephalopathy in these patients.

In addition to patients’ general medical status or diet, other factors can trigger or exacerbate HE. For example, alcoholic cirrhotic patients frequently experience elevated blood pressure in the portal vein transporting blood to the liver, which may lead to complications such as gastrointestinal bleeding and accumulation of fluid in the abdomen (a condition known as ascites). To treat these complications, physicians use a nonsurgical procedure known as trans-jugular intrahepatic stent shunt (TIPS), which redirects some of the blood flow around the liver. Following the procedure, blood bypasses the liver, as do the toxins contained in the blood (a process similar to the diversion of blood in patients with liver cirrhosis described in the introduction). Although TIPS leads to a lower blood pressure in the portal vein, thereby preventing gastrointestinal bleeding and ascites, it also can lead to HE because the blood is not detoxified. In fact, in up to 50 percent of patients treated with TIPS, the procedure results in new or worsening episodes of HE.

### Structural Brain Damage Associated With HE

HE associated with chronic liver failure (regardless of whether the liver disease is caused by alcohol or other factors) does not result in significant loss of nerve cells (i.e., neurons) in the brain or in other readily observable structural damage to the neurons. In contrast, liver failure causes significant changes to supporting brain cells called astrocytes. These large star-shaped cells are essential to the functioning of the central nervous system because they help maintain the proper composition of the fluid surrounding the neurons. For example, astrocytes take up brain chemicals (i.e., neurotransmitters) that are released by the neurons and mediate nerve signal transmission between cells; astrocytes also take up minerals (e.g., potassium) generated and secreted during the brain’s energy metabolism and remove neurotoxic substances. Some evidence suggests that a metabolic “cross-talk” (i.e., the release of substances by one cell type to stimulate another cell type) occurs between astrocytes and neurons which is essential to brain function.

**Figure 1:**
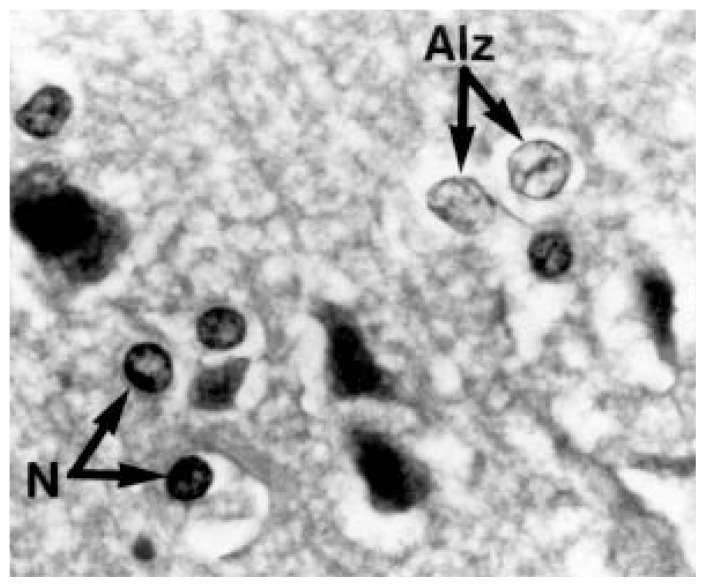
Brain cells called astrocytes from a 51-year-old alcoholic patient with cirrhosis who died in a coma (hepatic encephalopathy [HE]). The image shows both normal astrocytes (N), which have dark nuclei, and Alzheimer type II astrocytes (Alz), characteristic of HE, which have pale, enlarged nuclei. SOURCE: Modified from Butterworth 2002.

In patients with HE, the astrocytes adopt a characteristic morphologic feature known as Alzheimer type II astrocytosis, named after the neuropathologist who also gave his name to the neurodegenerative disease. These abnormal astrocytes, which usually are found in pairs or triplets, are characterized by their enlarged, glassy-looking nuclei (see figure 1). This glassy appearance is caused by the fact that the DNA and its associated proteins are not distributed throughout the nucleus but are confined to its edges. The nuclei of Alzheimer type II astrocytes also contain deposits of a molecule called glycogen, which serves as a storage form of the sugar glucose in cells but typically is not found in the nucleus; it is unknown whether these changes affect astrocyte function, however.

## Neuroimaging Helps Elucidate the Mechanisms Leading to HE

Researchers have gained a better understanding of the mechanisms and consequences of alcoholic liver disease in the brain by using neuroimaging and spectroscopic techniques that allow them to study the metabolism and functions of specific brain regions in living patients. Two neuroimaging techniques used extensively in the study of HE in alcoholic cirrhotic patients are positron emission tomography (PET) and magnetic resonance imaging (MRI). Studies using these techniques have confirmed the contributions of two neurotoxic substances, ammonia and manganese, to the development of HE.

### PET Establishes Ammonia’s Role

PET is a technique used to examine the metabolic activity of various body regions, including the brain. For these analyses, patients are injected with a molecule called a ligand, which normally may be metabolized in the body. The ligand molecule is labeled by the addition of a radioactive atom. Using sophisticated machines to detect the radioactive molecules in various tissues, investigators can monitor the transport of ligand molecules and their breakdown in the tissues, and thereby can determine the metabolic activities of those tissues. For example, researchers can measure glucose uptake, which occurs in all cells in the process of generating the energy those cells need, by administering a radioactive form of glucose called ^18^F-fluorodeoxyglucose (^18^FDG). Similarly, investigators can use radioactive water (^15^O-H_2_O) to monitor blood flow, radioactive ammonia (^13^N-NH_3_) to assess ammonia metabolism, and a wide range of radioactive compounds to study various molecules involved in the activities of neurotransmitters.

A variety of PET ligands have been used to assess the functions of various brain regions in patients with alcohol-induced cirrhosis. Studies using ^18^FDG have revealed that alcoholic cirrhotic patients with mild HE show decreases in glucose uptake in the region of the brain involved in controlling the “attention system” responsible for monitoring, for example, visual stimuli as well as in selecting appropriate responses to these stimuli (i.e., the anterior cingulate cortex) (Lockwood et al. 2002). These findings indicate that this brain region is less active in HE patients than in healthy people. Not surprisingly, the investigators also found that glucose utilization in the anterior cingulate cortex, as determined by ^18^FDG, correlated significantly with patients’ performance on neuropsychological tests involving attention-demanding tasks (i.e., patients with reduced glucose utilization exhibited poorer performance). Reduced glucose utilization in patients with HE was accompanied by corresponding decreases in blood flow in those brain structures.

**Figure 2:**
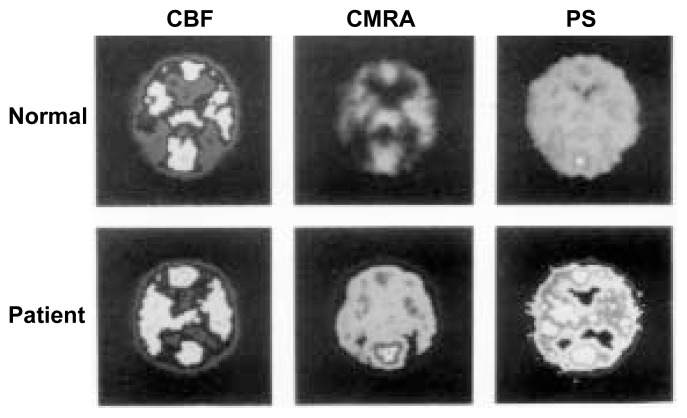
Positron emission tomography (PET) analyses of a healthy person and a 47-year-old alcoholic cirrhotic patient with mild hepatic encephalopathy. The blood flow through the brain (i.e., cerebral blood flow [CBF]) differs only minimally between the two subjects. However, the cerebral metabolic rate for ammonia (CMRA) and the permeability–surface area product (PS)—a measure of the extent to which ammonia can pass the blood–brain barrier and enter the brain—are significantly increased in the alcoholic patient, as indicated by the wider distribution and enhanced brightness of the light areas. SOURCE: Modified from Lockwood et al. 1991.

Other PET studies have assessed the levels of ammonia in the brain. As mentioned earlier, ammonia is one of the neurotoxic substances that the liver normally removes from the blood. PET analyses using radioactive ammonia in cirrhotic patients with mild HE have revealed significant increases in ammonia uptake and metabolism in the brain (see figure 2) (Lockwood et al. 1991). These investigators found that, in particular, a measure of the extent to which ammonia can enter the brain from the general circulation^3^—a variable called the permeability–surface area product (PS)—increases as cirrhotic patients start to develop HE. When the PS increases, a greater proportion of the ammonia present in the general circulation can enter the brain. Increases in the PS explain why severe HE can be found in alcoholic cirrhotic patients even if the ammonia levels in the blood are not greatly elevated: In these cases, a higher proportion of the ammonia in the blood can enter the brain and cause damage there. Studies have found that in patients with advanced stages of encephalopathy resulting from chronic liver failure, ammonia levels in the brain may increase more than twentyfold (Butterworth 2002).

Ammonia is extremely toxic to the brain, affecting both neurons and astrocytes. For example, ammonia has deleterious effects on nerve signal transmission mediated by numerous neurotransmitter systems (Szerb and Butterworth 1992) as well as on the brain’s energy metabolism. In addition, ammonia can alter the expression^4^ of various genes encoding key brain proteins involved in the brain cells’ energy production, structure, and cell-to-cell interactions. (For more information on the specific genes affected by ammonia, see the sidebar “Altered Gene Expression in the Brain Resulting From Alcoholic Liver Disease.”) These alterations in gene expression may account for some of the changes in neurotransmitter activity and astrocyte structure observed in HE patients.

**Altered Gene Expression in the Brain Resulting From Alcoholic Liver Disease**

One of the main causes of brain dysfunction in patients with hepatic encephalopathy (HE) is the accumulation of ammonia in the blood, which the patient’s liver, damaged by alcoholic liver disease, cannot remove. Molecular biological techniques have allowed researchers new insights into how HE develops, including the specific effects of ammonia. These analyses have found that, among other effects, ammonia alters the expression of certain genes—that is, the rates at which the genetic information contained in DNA is converted into the final protein products encoded by those genes. Molecular approaches have enabled investigators to identify some of the genes affected in patients with HE (Butterworth 1998). These genes code for key brain proteins that are essential to the cells’ energy production, structure, and interactions with other cells, including the following proteins:

- A specific type of *monoamine oxidase* called MAO-A, an enzyme responsible for the metabolism of neurotransmitters called monoamines. These compounds include the neurotransmitter serotonin, which is involved in the regulation of mood and sleep.
- *The mitochondrial (peripheral-type) benzodiazepine receptor,* a receptor protein located on the membranes surrounding small cell structures called mitochondria, which serve as the cell’s energy factories. This receptor helps facilitate the uptake of cholesterol into the mitochondria, which then is converted into a family of compounds known as neurosteroids (Desjardins and Butterworth 2002). Neurosteroids can excite or inhibit other neighboring nerve cells, thereby influencing nerve signal transmission. The brains of patients with alcoholic liver disease show increased production of the neurosteroid allopregnanolone, a potent neuroinhibitory compound that could contribute to the development of HE in alcoholic liver disease. This increased production probably results from enhanced cholesterol uptake mediated by the benzodiazepine receptor.
- A nerve cell–specific form of the enzyme *nitric oxide synthase,* which produces nitric oxide, a highly reactive molecule (i.e., a free radical) that plays a role in various cellular processes, including cell-to-cell communication and the widening (i.e., dilation) of blood vessels (Raghavendra Rao et al. 1997). Excess levels of nitric oxide, however, are toxic to the cells and can contribute to a cellular state called oxidative stress, which is characterized by the presence of excess free radicals and/or a lack of the compounds that remove these free radicals (i.e., antioxidants). Oxidative stress can cause cell damage and has been implicated in alcoholic liver disease. Increased expression of nitric oxide synthase in the brains of patients with chronic liver failure has been linked to excess nitric oxide production, which in turn leads to oxidative stress and cellular dysfunction.
- A protein called *glial fibrillary acidic protein (GFAP),* which is essential for maintaining the structure of astrocytes (Bélanger et al. 2002). Chronic liver failure or ammonia exposure results in decreased expression of GFAP, which is associated with the presence of Alzheimer type II astrocytes that are characteristic of HE.

—*Roger F. Butterworth*

ReferencesBélangerMDesjardinsPChatauretNButterworthRFLoss of expression of glial fibrillary acidic protein in acute hyper-ammonemiaNeurochemistry International412–315516020021202061510.1016/s0197-0186(02)00037-2ButterworthRFAlterations of neurotransmitter-related gene expression in human and experimental portal-systemic encephalopathyMetabolic Brain Disease1333734919981020682510.1023/a:1020641009971DesjardinsPButterworthRFThe “peripheral-type” benzodiazepine (omega 3) receptor in hyperammonemic disordersNeurochemistry International4110911420021202061110.1016/s0197-0186(02)00031-1Raghavendra RaoVLAudetRMButterworthRFIncreased neuronal nitric oxide synthase expression in brain following portacaval anastomosisBrain Research7651691721997931040910.1016/s0006-8993(97)00652-5

Ammonia normally is removed from the blood in the liver by a series of chemical reactions called the urea cycle. During these sequential reactions, ammonia is converted into urea, which is excreted in the urine. The brain, however, lacks an effective urea cycle and therefore has only a limited capacity to remove any ammonia that enters the tissue from the blood because of the increased PS. The only way to eliminate any ammonia that has reached the brain cells is through a reaction mediated by an enzyme called glutamine synthetase, which combines a molecule of the amino acid glutamate with a molecule of ammonia to form the amino acid glutamine. The relevance of this reaction was confirmed in neuroimaging analyses measuring glutamine levels in the brains of alcoholic patients with HE. These studies found that the amounts of glutamine formed in the brain correlated with the severity of HE, confirming that the brain is exposed to elevated levels of ammonia (Lockwood et al. 1997). However, glutamine synthetase is present only in astrocytes, not in neurons, and cannot remove all the ammonia that enters the brain. As a result, neurons are virtually defenseless against increased ammonia concentrations and therefore are most likely to exhibit impaired function indicative of ammonia-related damage.

### MRI Identifies Signal Hyperintensities That May Result From Manganese Deposits

MRI technology is based on the fact that when the body is exposed to radio waves in the presence of a strong magnetic field, the nuclei of certain atoms emit signals as they align and realign in the magnetic field. These signals can be detected by a scanning device and converted into three-dimensional images of specific structures of the body (e.g., brain structures). MRI scanners generally form images based on signals emitted by hydrogen atoms, the most abundant element in biological tissues. Because most of the hydrogen in the body is in the form of water, MRI images differentiate brain structures based on their water content. However, elements other than hydrogen, including manganese, also can emit signals that are detected by MRI, as discussed below.

**Figure 3:**
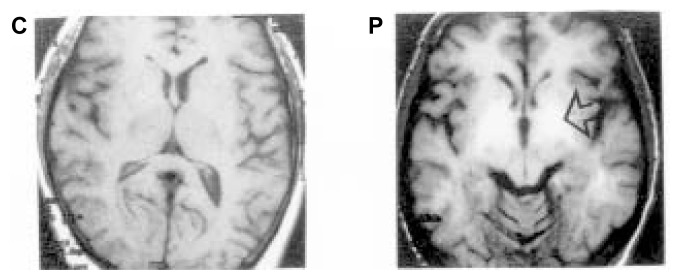
Magnetic resonance imaging (MRI) of a healthy control subject (C) and an alcoholic cirrhotic patient of the same age (P). In the alcoholic patient, abnormally intense signals (arrow) are detected on both sides of the brain in a region called the globus pallidus. This phenomenon has been attributed to deposits of manganese in this brain area. SOURCE: Lockwood et al. 1997.

When MRI analyses are performed on alcoholics with cirrhosis, more than 80 percent of the patients show regions of abnormally high signal intensity (i.e., signal hyperintensities) in both the right and left halves of the brain and concentrated in an area that is involved in the control of motor function (i.e., the globus pallidus) (see figure 3) (Lockwood et al. 1997; Spahr et al. 2000). The elevation in intensity of these signals correlates with the presence of certain signs and symptoms of impaired motor function that are found, for example, in patients with Parkinson’s disease (e.g., rigidity, abnormally diminished motor activity, and tremors) and also are present in HE patients (Spahr et al. 2000). However, the intensity of the MRI images does not correlate with the patients’ performance on tests assessing global encephalopathy and cognitive functioning.

Increasing evidence suggests that the intense MRI signals in the globus pallidus of cirrhotic alcoholics are caused by deposits of a paramagnetic substance, probably manganese, in that region (Lockwood et al. 1997). Manganese normally is eliminated by the joint actions of the liver, gallbladder, and bile ducts (i.e., the hepatobiliary system). In patients with chronic liver failure, manganese concentrations in the blood increase, and the metal can enter the brain and be deposited in the globus pallidus and associated brain structures (i.e., the basal ganglia). Studies using brain tissue from alcoholic cirrhotic patients who died from HE revealed up to seven times more manganese in the globus pallidus compared with normal levels (Butterworth et al. 1995). Manganese is known to be neurotoxic, particularly affecting the actions of certain proteins (i.e., receptors) that interact with the neurotransmitter dopamine. Consistent with this finding, researchers found altered dopamine receptors in the brains of alcoholic cirrhotic patients who died in a hepatic coma (Mousseau et al. 1993). In addition, manganese induces Alzheimer type II changes in astrocytes. Taken together, these observations suggest that deposits of manganese in the globus pallidus resulting from chronic liver failure probably cause the motor symptoms and structural changes of astrocytes that are characteristic of HE.

Amounts of other metals whose chemical properties are similar to those of manganese are not substantially elevated in the brains of patients with chronic liver failure, ruling out those metals as causes of HE and its symptoms.

## Management of Patients With HE

Researchers and clinicians are exploring a variety of approaches for preventing HE in patients with alcohol-induced chronic liver failure or for ameliorating its consequences. These approaches involve strategies to lower the levels of ammonia in the blood, medications to counteract ammonia’s neurotoxic effects, devices to improve liver function, and liver transplantation.

### Ammonia-Lowering Strategies

In patients with cirrhosis, HE is frequently triggered by conditions that cause an increase in circulating ammonia, including gastrointestinal bleeding or a diet that contains high amounts of protein (which generates ammonia when broken down). Because levels of ammonia in the blood and brain are elevated in these patients, the most popular strategies currently used to manage and treat HE involve reducing ammonia production or increasing ammonia metabolism (i.e., the conversion of ammonia into harmless molecules) outside the brain.

Traditional ammonia-lowering strategies include the use of certain sugar molecules that are not absorbed into the body (e.g., lactulose) or certain antibiotics (e.g., neomycin), both of which reduce the production of ammonia in the gastrointestinal tract. To increase ammonia metabolism, one can administer an agent called L-ornithine L-aspartate to patients, which enhances the natural process by which ammonia is incorporated into the amino acid glutamine in the skeletal muscle; this agent also optimizes the residual urea synthesis in the patient’s cirrhotic liver. An earlier approach to lowering ammonia levels—that is, seriously limiting the protein intake of patients with alcoholic cirrhosis—is no longer recommended because it can lead to a reduction in the patient’s muscle mass. Maintaining muscle mass is important because, as mentioned above, a chemical reaction that removes free ammonia from the blood by incorporating it into glutamine can occur in the skeletal muscle.

### Neuropharmacological Approaches

Other possible strategies for managing HE involve using neuroactive drugs to counteract ammonia’s harmful effects on neurotransmitter systems in the brain. This treatment approach still is in its infancy, however, because researchers have not yet identified the precise nature of the neurotransmitter systems that either play a role in the development of HE or are affected by the condition. Some studies have reported occasional beneficial effects when the dopamine neurotransmitter system was stimulated by agents such as L-dopa or bromocriptine, but these approaches have not gained wide acceptance. Likewise, a compound called flumazenil that inhibits the actions of sedative agents (i.e., is a benzodiazepine antagonist) initially was reported to be beneficial, having an awakening effect on stuporous and comatose alcoholic cirrhotic patients (Pomier Layrargues et al. 1994). However, researchers now think that the beneficial action of flumazenil resulted from its activity as an antidote to the benzodiazepine medications frequently prescribed to alcoholic cirrhotic patients as part of an endoscopic evaluation or as a sedative. Thus, flumazenil may correct benzodiazepine-induced coma in these patients rather than treat HE per se.

### Liver-Assist Devices

A great deal of attention currently is focused on the production of “artificial livers,” or liver-assist devices. In general, these devices are composed of small columns filled with functional hepatocytes, a protein called albumin, or charcoal, or combinations thereof. The patient’s plasma is circulated through the columns to remove toxins in a manner similar to dialysis treatment of patients with kidney failure. Initial studies using an albumin system yielded particularly promising results: Patients treated with this approach showed less ammonia circulating in the blood as well as amelioration of their HE symptoms (Mitzner and Williams 2003).

### Liver Transplantation

Liver transplantation is the ultimate treatment for alcoholic cirrhotic patients with end-stage chronic liver failure. In general, implantation of a new liver results in normalization of blood ammonia concentrations as well as in significant improvements in cognitive function in these patients (Arria et al. 1991), clearly confirming that HE is a major contributor to the cognitive dysfunction found in alcoholic patients with significant liver disease. Liver transplantation also eliminates the MRI signal hyperintensities that result from manganese deposits found in the brains of patients with HE. However, the MRI signal hyperintensities may take several months to resolve (Pujol et al. 1993), suggesting that manganese is removed slowly from the brains of these patients.

## Summary

HE is a major neuropsychiatric complication of alcoholic liver disease and an important contributor to the cognitive dysfunction found in chronic alcoholic patients. HE is caused in part by the accumulation in the brain of neurotoxic substances such as ammonia and manganese, which normally are removed by the hepatobiliary system. Increased brain concentrations of ammonia alter the expression of genes that encode important brain proteins responsible for regulating neurotransmitters; higher levels of ammonia also alter the structure of brain cells called astrocytes. Manganese deposited in basal ganglia structures (particularly the globus pallidus) in patients with alcoholic cirrhosis leads to impaired motor function and the appearance of distinct, abnormally intense signals, which can be detected by MRI. Prevention and treatment of HE in alcoholic cirrhotic patients continue to rely on strategies aimed at lowering blood ammonia concentrations. Current research is focused on identifying neurological and biochemical mechanisms underlying HE as well as pharmacotherapies that can address these mechanisms, and on creating liver-assist devices aimed at removing toxins from the blood. Liver transplantation is used in chronic alcoholic patients with end-stage liver failure.

## Footnotes

1 The term “encephalopathy” refers to any abnormal condition in the structure or function of the brain, especially chronic, destructive, or degenerative conditions.

2 The serum is the clear portion of the blood that remains after blood cells, platelets, and blood-clotting molecules have been removed.

3 The brain is largely separated from the general circulation by the blood–brain barrier, a property of the blood vessels in the brain that prevents the passage of many molecules from the blood into the brain tissue. As a result, only selected substances can enter the brain from the blood. This feature protects the brain cells from exposure to many toxic substances or disease-causing organisms; however, some molecules (e.g., ammonia) can pass the blood–brain barrier to a certain extent.

4 The term “gene expression” refers to the entire process of converting the genetic information encoded in a gene into a protein product.
